# Survival rate of eukaryotic cells following electrophoretic nanoinjection

**DOI:** 10.1038/srep41277

**Published:** 2017-01-25

**Authors:** Matthias Simonis, Wolfgang Hübner, Alice Wilking, Thomas Huser, Simon Hennig

**Affiliations:** 1 Biomolecular Photonics, Department of Physics, University of Bielefeld, Universitätsstr. 25, 33615 Bielefeld Germany; 2 Department of Internal Medicine and NSF Center for Biophotonics, University of California, Davis, Sacramento, CA 95817, USA

## Abstract

Insertion of foreign molecules such as functionalized fluorescent probes, antibodies, or plasmid DNA to living cells requires overcoming the plasma membrane barrier without harming the cell during the staining process. Many techniques such as electroporation, lipofection or microinjection have been developed to overcome the cellular plasma membrane, but they all result in reduced cell viability. A novel approach is the injection of cells with a nanopipette and using electrophoretic forces for the delivery of molecules. The tip size of these pipettes is approximately ten times smaller than typical microinjection pipettes and rather than pressure pulses as delivery method, moderate DC electric fields are used to drive charged molecules out of the tip. Here, we show that this approach leads to a significantly higher survival rate of nanoinjected cells and that injection with nanopipettes has a significantly lower impact on the proliferation behavior of injected cells. Thus, we propose that injection with nanopipettes using electrophoretic delivery is an excellent alternative when working with valuable and rare living cells, such as primary cells or stem cells.

To deliver foreign molecules to the cytoplasm of living cells, one has to distinguish single cell delivery techniques from ensemble methods such as electroporation^1^, chemical permeabilization^2^ or glass bead delivery^3^. These are, in most cases, used on large numbers of cells in culture and it is commonly accepted that a significant number of these cells (up to 50%) will either not survive this process^4^ or that the cell cycle of a significant number of cells is disrupted^5^. Newer techniques such as cell squeezing^6^,^7^, or massive parallel delivery with light pulses^8^ enable more control over the process but are still of a stochastic nature. These stochastic processes lack the ability to specifically address single cells.

Single cell delivery methods are mainly based on the physical injection of cells with small glass pipettes, but also non-penetrating pipette-based methods are known^9^,^10^, exploiting photothermal effects to overcome the plasma membrane of living cells. Injection-based single-cell methods offer a valid alternative to stochastic delivery methods. A large number of injection methods have been developed, ranging from charged lance injectors^11^ over AFM-based injection methods^12^ to classic microinjection with injection volumes in the nanoliter regime^13^,^14^. Microinjection is widely used in biological research for a variety of experiments and different samples from single cells to small organisms have successfully been utilized with this technique^15^,^16^,^17^,^18^. For this purpose, a glass capillary is first pulled from a cylindrical quartz or borosilicate blank to result in a fine tip of typically 0.5–1.0 μm in diameter. Micromanipulators are then used to direct these tips to their target. The process resulting in the injection of small liquid volumes that contain the biomolecules of interest is mostly pressure-driven. The injection success rate and the survival rates of injected cells depend strongly on the skills of the operator and the specific cell type as well as the amount of the injected volume. A wide range of survival rates varying between 9% to 56% (human blood stem cells^19^, up to 49% to 82%) was reported^19^,^20^. Wang *et al*. used a semi-automated microinjection system and were able to improve the short term survival rate to above 95% but provide no information about long-time viability. The fairly low and widespread injection success rates of microinjection applied to cultured cells with a typical diameter of 10–20 μm is mostly due to the lack of accurate position feedback from the micropipette. Based purely on optical observation, the height of adherent cells is difficult to estimate and often the experimentator cannot determine whether the tip of the micropipette is just inside the cell or might have already reached the substrate surface. Multiple injection attempts are often necessary which further decreases the viability of cells.

Seemingly, a critical determinator of cell viability appears to be the size of the injection probe relative to the cell size. Thus, developing smaller delivery devices such as nanotubes or nanowires attached to AFM cantilevers^21^ or micropipettes^22^ appears reasonable. All these devices use significantly smaller injection probes, but still don’t offer an adequate feedback about the approach, penetration and injection process for the user. Furthermore, these methods become relatively complex to use and the delivery probes are tricky to fabricate compared to the simple process of pulling a glass pipette with a similarly small tip diameter.

Hansma *et al*. (1989)^23^ were the first to show that the nanopipette setup used in scanning ion conductance microscopy (SICM) makes it possible to obtain precise feedback of the pipette position relative to a surface. A modification of this scanning device made it possible to deposit molecules onto single cells^24^. Additionally, due to (di-)electrophoretic forces, the deposition of molecules could be precisely controlled^25^. The next step was to inject molecules directly into cells, overcoming the cell membrane similar to microinjection, except that the tips are now about ten times smaller in diameter and the delivery is driven by electrophoretic forces, using typical injection voltages of ± 20 V^26^. Compared to microinjection, the use of electrophoretic forces instead of pressure driven liquid transfer and the smaller pipette diameters should have a significantly reduced impact on the overall volume displacement inside the cell, as well as the disrupted area of the membrane and cytoplasm of the cell, and therefore its viability. Recent experiments with nanoinjection have shown that it is also possible to deliver molecules with low voltages of 50–100 mV, making it possible to monitor the delivery of fluorescent molecules with single molecule sensitivity. Injection of a wide range of different functionalized fluorescent probes into cells and cell compartments was possible while still having excellent position control^27^.

Here, we show that nanoinjection results in a *long-term cell viability* of 92% following the electrophoretic injection process with a 100 nm diameter nanopipette. We minimize the damage inflicted to the cells by piezo-actuated approach and control the injection process by feedback based on monitoring and adjusting the ionic current on the fly. Nanopipettes are easy to fabricate using a laser-heated pulling process which allows for quick adjustments and optimization during an experiment. To show that cell viability strongly depends on the size of the pipette, we additionally used standard 500 nm microinjection tips under the same conditions leading to a long-term survival rate of 40% after 24 hours. Additionally, we found that the duration and magnitude of the generated electric field in the direct vicinity of the pipette during a typical nanoinjection process appears to have no effect on the cell’s health. Furthermore, we show that even the direct injection of molecules into the nucleus using a 100 nm nanopipette does not significantly affect cell health.

## Results and Discussion

To achieve reliable statistics for the survival rate of nanoinjected cells, we injected a total of 239 cells with a cell impermeant dextran construct labeled with fluorophores (Dextran - Alexa Fluor 647, DAF), which enables direct monitoring of the injection process and the subsequent observation of the cells for extended time periods. Since we suspected that the survival of cells correlates directly with the diameter of the tip, we compared the effects of using two different tip diameters (100 nm and 500 nm). A tip diameter of 100 nm represents the typical diameter of a nanopipette (see Figure S1), while a diameter of 500 nm represents the typical diameter of microinjection pipettes. The injection of single cells was carried out as described in Materials & Methods.

All percentages reported from here on have already been corrected with regard to a control population of 184 cells that were located directly next to the injected cells and therefore investigated under the exact same culture conditions. As the mortality of cells, either by natural causes *M*_*cont*_ (which was measured to be 6% (N = 184) under the conditions used in our experiments) or after the injection process *M*_*inj*_(*P*_*inj*_) are independent of each other, the total probability of a cell to die by the injection process is 
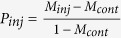
.

**Figure 1:**
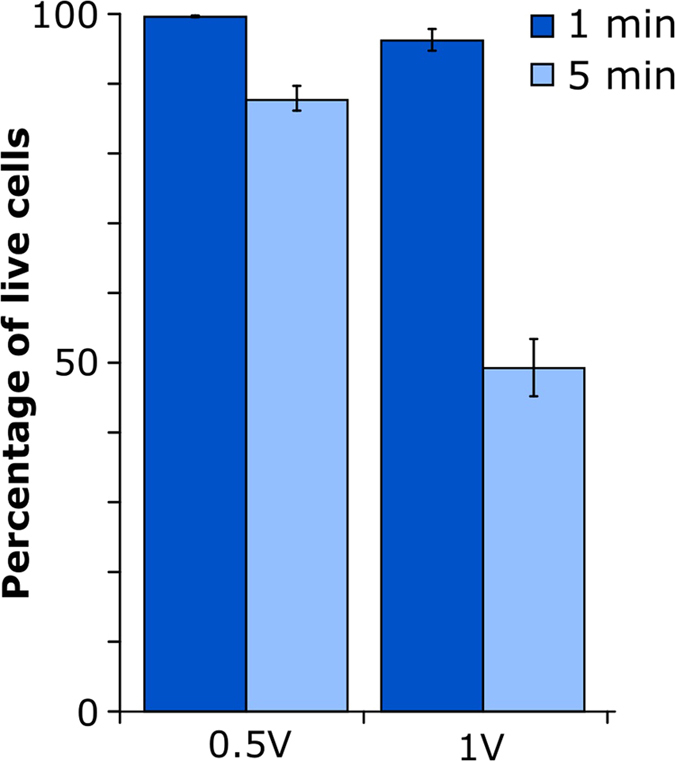
Statistics of cell viability 24 hours after injection with two different voltages and timings. The ‘injection’ was carried out with nanopipettes (100 nm), loaded only with PBS (i.e. without fluorophores). Approached and inserted into the cytoplasm of single living cells, the pipette was left inside each cell either for 1 min. or 5 min. The applied voltage was set to either 1 V or 500 mV. Afterwards the ‘injected’ cells are observed for 24 hours. The result shows that for 1 min. ‘injection’ time the voltage has no impact on viability with 94% (N = 35) for 1 V and 97% (N = 32) for 500 mV. The original data yields equal or higher viability than the control and hence is considered 100%. After 5 min. however the difference clearly shows. Only 49% (N = 26) of cells treated with 1 V survived the ‘injection’ whereas the viability at 500 mV is almost 30% higher at 85% (N = 17). For experiment details see Table S1.

The voltage applied during the electrophoretic injection process could also have an additional negative effect on cell health. To evaluate this effect, we first measured the survival rate for long injection times and for high injection voltages. We injected living cells with a nanopipette (100 nm diameter) loaded only with PBS (i.e. without fluorophores). To simulate the injection process we left the pipette inside the cell for 1 min. and for 5 min. after approach and penetration (the typical injection times for later experiments ranged between 5 to 10 seconds) and applied voltages of 1 V and 500 mV, respectively. We investigated the same ‘injected’ cells 24 h later by wide-field microscopy to determine whether they were alive, or dead. We found, that 1 min. of injection time has a negligible influence on the cells’ health independent of the applied voltage (Fig. 1). The cell death rate after 24 h was in the range of the expected control mortality. On the other hand, longer exposure to the tip (5 min.), as well as applying a higher voltage (1 V) had a significant impact on cell health. After 24 h the difference in injection voltage lead to survival rates of 87% (500 mV, 5 min.) and 47% (1 V, 5 min.). To avoid these detrimental effects, all experiment reported below were conducted using nanoinjection exposure times of well below 1 min. According to this, the nanoinjection process should then be mostly independent of the voltage used for the injection process and it can consequently be assumed, that the cell survival is indeed just dependent on the size of the injection probe.

**Figure 2:**
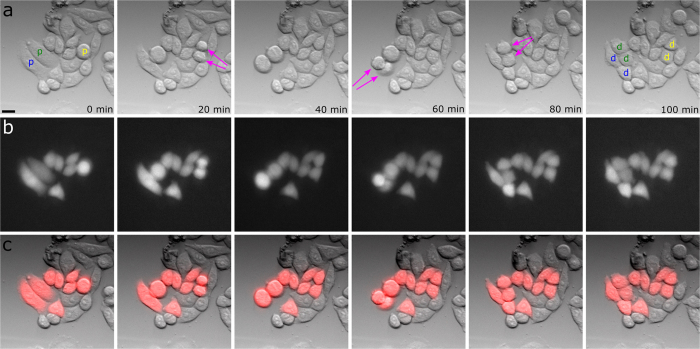
Time-lapse images of living U2OS cells injected with DAF. (**a**) Whitelight, (**b**) wide-field fluorescence and (**c**) an overlay of both. “p” marks parental cells, the arrows indicate a proliferation event and “d” is for the corresponding daughter cells. Images were acquired every 20 min, using the intensity setting 5% on the DeltaVision Elite at an integration time of 200 ms and 10 ms for the red (632 nm) and whitelight excitation respectively. Scale bar, 20 μm. The full movie can be seen in the Supplementary information (Movie S1).

In an attempt to obtain significant statistics about the fate of single cells following treatment with different pipette sizes, we injected single cells within an ensemble of cells plated on gridded glass-bottom culture dishes. The cells were tracked for a period of 24 h. A sample time-lapse series of single injected cells over a period of 100 min. shows the injected cells during mitosis, demonstrating normal behavior of the cells and a presumably healthy cell cycle (Fig. 2). White light and fluorescence images were taken every 20 min.

**Figure 3:**
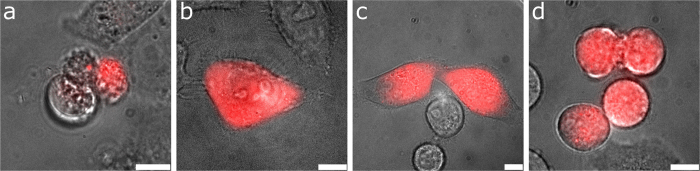
Overlay of the wide-field fluorescence and whitelight images of single U2OS cells. The images were acquired 24 hours after nanoinjection of DAF. The cell morphology and proliferation were used to assess cellular health. (**a**) Dead or dying cell; residues of the dye are still visible. (**b**) Healthy cell with normal morphology. (**c**) Two healthy cells with dye inside them that originated from one injected cell (mitosis). (**d**) Four cells just finishing mitosis, the upper two in the image are still attached to each other. The images were acquired with common wide-field excitation intensities at an integration time of 100 ms. Scale bars, 10 μm.

**Figure 4:**
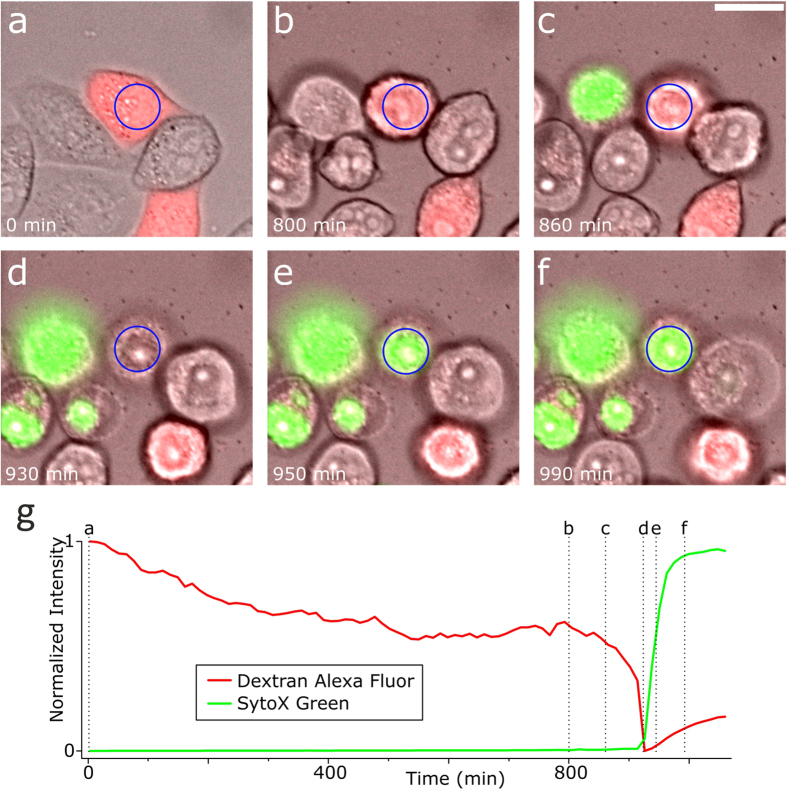
Time-lapse fluorescence-/whitelight-imaging of a cell injected with DAF in a 100 nM SytoX Green Nucleic Acid Stain (SXG) environment. The cells were kept at 37 °C in culture medium. Both dyes are impermeant to the intact cellular membrane of living cells. DAF does not bind to a specific target inside the cell whereas SXG will label the DNA after entering the cell. (**a**–**f**) Overlay of whitelight, green and red fluorescent images of a single dying U2OS cell (blue circle). (**a**) Healthy cell. The injected DAF is visible within the injected cell (red fluorescence). (**b**–**d**) During cell death, the DAF fluorescence will decrease as the DAF molecules leave the cell due to the corrupted cellular membrane. Simultaneously, the SXG molecules begin to enter the cell, indicated by the increased green fluorescence inside the cell. (**e**,**f**) The unbound fluorophores diffuse out of the cell while SXG binds to the DNA and creates a strong green fluorescent signal inside the nucleus. (**g**) Normalized intensities of red and green fluorescence of the area indicated by the blue circle. The decrease in intensity from a) to b) is likely due to photobleaching and the dye being slowly discarded from the cell. Images were acquired every 10 min using the intensity settings 5%, 2% and 5% on the DeltaVision Elite at an integration time of 250 ms, 80 ms and 10 ms for the red (632 nm), green (475 nm) and whitelight excitation respectively. Scale bar, 20 μm.

For statistical analysis, we determined the number of living and dead cells as well as the proliferation rate of the treated cells within the field of view of a wide-field imaging system. As mentioned previously, we chose DAF for injection. These probes allowed us to employ fluorescence microscopy to monitor the successful delivery of molecules to cells because they have been reported to be nontoxic and traceable over many cell cycles^28^. Statistical information about injected cells was obtained by injecting the dextran-dye conjugate into the cytoplasm or the nucleus of single cells with subsequent review of the treated cells within and after 24 hours. The gridded glass bottom enabled us to locate the cells over extended periods of time. The injected cells were far enough apart to prevent mix ups of the treated cells. The findings can be divided into three different outcomes:

1: The cell died within 24 h after nanoinjection. When the cell dies, the cellular membrane becomes disrupted and the injected dye diffuses out of the cell. If an initially present fluorescent signal disappears, the cell is presumably dead. In some cases the dead cell leaves some fluorescent residues behind (Fig. 3a) but these can be distinguished from healthy cells by their morphology and are not counted as false positives. To show that injected DAF can indeed be used as a dead cell indicator, we added 100 nM SYTOX Green nucleic acid stain (SXG), to the cell medium in a different experiment and left the injected cells sitting at room temperature and under ambient atmosphere. The results (Fig. 4) show that when SXG accumulates inside the nucleus of the cell, DAF begins to diffuse out of the cell. Thus, it is possible to mark the same time of cell death with these different methods.

2: If the cell is still alive, it can easily be found by its fluorescence. This implies that the cell membrane stayed intact throughout the duration of the experiment and the cell morphology looks normal (Fig. 3b). During mitosis the cell can somewhat appear rounded and is still counted as alive and well (Fig. 3d).

3: If the cell has proliferated within the past 24 hours, instead of one cell we found two (Fig. 3c) or in some cases even four cells (Fig. 3d) exhibiting intracellular fluorescence.

**Figure 5:**
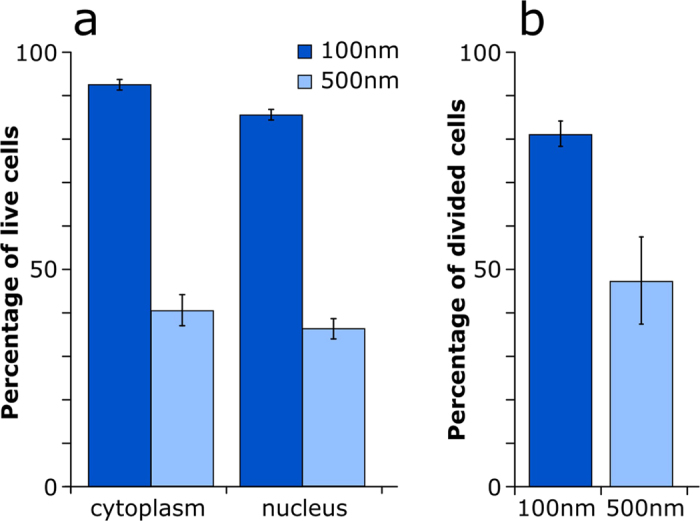
Statistics of cell status 24 hours after the injection of DAF with a 100 nm and 500 nm pipette into the nucleus and cytoplasm. Prior to injection, the cells were cultivated on μ-Dishes for at least 24 h. For the injection process which took all in all less than an hour, the cell medium was substituted with pre-warmed PBS for a better approach feedback and afterwards again replaced with pre-warmed DMEM and kept in an incubator at 37 °C, 5% CO_2_. (**a**) shows viability after the different experiments one day later. For cytoplasmic injection it is 92% (N = 68) for 100 nm and 40% (N = 50) for 500 nm. Injection into the cells nucleus leads to an insignificantly lower viability of 85% (N = 71) and 36% (N = 50) respectively. (**b**) Comparison of the surviving cells only, with regard to the proliferation percentage. Either we found them alone after 24 hours or we were able to see daughter cells containing the injected dextran in both cells, indicating the proliferation. 81% (N = 116) of the nanoinjected cells divided, whereas only 47% (N = 36) of the cells treated with the 500 nm pipette showed proliferation.

The examination of the treated cell cultures with a total of 68 single injected cells 24 hours after injection of DAF into the cytoplasm, reveals a total long-term survival rate of 92% using the 100 nm nanopipette. In contrast, cells injected with the 500 nm diameter pipette showed a long-term survival rate of 40% for a total of 50 injected cells. Injection of DAF directly into the nucleus lead to slightly lower survival rates: 85% and 36% respectively for a total of 121 cells. (Fig. 5a). In addition to the survival rate, all surviving cells were further checked for their proliferation behavior. The percentage of injected cells which have divided within 24 h after the injection process, was determined to be 81% for 116 cells surviving the treatment with 100 nm pipettes. Out of the 36 surviving cells treated with the 500 nm diameter pipette only 47% did divide (Fig. 5b). This shows that not only twice as many cells survive the penetration by the smaller 100 nm pipette but that the remaining vital cells are showing a more natural cell cycle as the control cells which all proliferated within 24 hours. This knowledge is crucial for further experiments with injected cells, because after penetration and manipulation of single cells the affected cells should behave as natural as possible.

In conclusion we have demonstrated that the size of the nanoinjection pipette tip has a significant influence on cell viability. We found that injection of molecules with a large tip diameter of 500 nm not only lowers the overall survival rate significantly, but also affects the cell cycle of otherwise healthy looking cells. The injection of fluorescent probes using smaller tip diameter of 100 nm lead to an overall control corrected survival rate of 92%. Furthermore, the surviving cells within the quantity of cells injected with 100 nm pipettes displayed a more normal proliferation behavior which is very important for long-time live-cell studies. For experiments using electrophoretic delivery of probes through nanopipettes, we found that extended injection with high voltages above 500 mV and 5 min. exposure time lowers the overall survival rate. The results yield good prospects for future work with nanoinjection experiments, using more fragile and valuable cell lines or primary cells in combination with nanopipettes of a diameter of 100 nm or less. Further reducing the nanopipette tip size will certainly increase the survival rate of the injected cell. Typical nanopipettes used e.g. for SNOM probes reached a tip diameter of approx. 30 nm^29^. In theory, the achievable tip diameter is limited to approx. 10 nm, using quartz glass capillaries (Sutter Instrument, P-2000 Operation Manual). At this point, the pipette diameters come close to the diameter of the injected molecule and the injection of molecules cannot be ensured. Hence, injection techniques based on nanopipettes with a tip diameter of 100 nm appear to be an appropriate tool for experiments involving the injection of most macromolecules into single living cells.

## Materials and Methods

### Cells

Human bone osteosarcoma cells (U2OS) were grown in Dulbecco’s Modified Eagle Medium (DMEM) with 5% fetal bovine serum (FBS) added and cultivated at 37 °C in a humidified 5% CO_2_ atmosphere. For all experiments, cells were transferred to 35 mm gridded culture dishes (μDish, Ibidi) at the desired density and given at least 24 h to settle down.

### Fluorophores

Dextran, Alexa Fluor 647; 10,000 MW, Anionic, Fixable (DAF) (Thermo Fisher Scientific, D22914) SYTOX Green Nucleic Acid Stain (SXG) (Thermo Fisher Scientific, S7020).

### Pipettes and electrodes

To fabricate nanopipettes, borosilicate glass filaments (outer diameter 1.00 mm, inner diameter 0.58 mm) were pulled to an outer diameter of approx. 100 nm. The P2000 puller (Sutter Instruments) used the following program:

Line1: HEAT:350, FIL:3, VEL:30, DEL:220, PULL:0

Line2: HEAT:330, FIL:2, VEL:27, DEL:180, PULL:250

For comparison, microinjection pipettes (Femtotips, Eppendorf) with an inner diameter of 0.5 μm were purchased. Both pipette types were filled from the back with a microloader (Eppendorf).

### Setup

For injection of cells a custom built setup was used consisting of an inverted IX71 microscope (Olympus, Tokyo, Japan) with a TIRF oil immersion objective (APO-N 1,49 NA/60x, Olympus) in combination with a xyz-piezo element (MCL Nano-PDQ375HS, Mad City Labs, Madison WI, USA). A custom build heat plate was used to maintain the needed 37 °C for working with live cells. Manual alignment of pipettes was accomplished by a manual xyz-control-unit (M-562 XYZ ULTRAlign, Newport, Irvine CA, USA). Chlorinated electrodes were used (silver wire, Nr. 786500, A-M Systems Inc. USA) in combination with a microelectrode amplifier (Axopatch200B, Molecular Devices, Union City, USA). An ArKr^+^ laser (70C-Spectrum, Coherent, Santa Clara, CA, USA) and a diode laser (i-Beam smart, TOPTICA Photonics, Germany) enabled excitation with 488 and 643 nm. Images were acquired by an EMCCD camera (iXON + , Andor, Belfast, Ireland). A scheme of the injection-setup is depicted in Figure S2.

### Additional imaging

Additional live-cell imaging was carried out with a DeltaVision Elite Imaging System (GE Healthcare, USA). Equipped with a live-chamber it was possible to maintain 37 °C with minimal deviations and also provide a humidified 5% CO_2_ atmosphere. Data were collected with a 20× air objective.

### Cell treatment and nanoinjection

The nanoinjection process was conducted according to (Hennig *et al*.^26^). Prior to injection, the cell culture medium was replaced with pre-warmed PBS to get a more precise ionic current signal as the pipette approaches the cell. After the cell dish was placed on the microscope, the pipette was filled with 10 μl of 1 μM DAF solution and fixed in the holder. The focal plane then was set approx. 20 μm above the cells to avoid breaking the pipette tip during coarse adjustments. After the tip was visible and set to match the focal plane manually, the pipette was positioned laterally above a cell of interest, either directly over the nucleus for a nuclear injection or above another region for cytoplasmic injection. The electrodes were placed inside the cell medium and the pipette and a potential of approx. −100 mV was applied. Now, the software controlled approach was started and while the ionic current was monitored the tip approached the cell. For cytoplasmic injection we stopped at the first, for nuclear injection at the second drop of current decrease, so that the cell was penetrated only as far as needed. To monitor the staining process, red excitation light was turned on. The voltage was then reversed and increased to about 1 V for 5 to 10 s for injection with nanopipettes and 1 s for injection with micropipettes as this was sufficient to label the cell. After retraction of the tip, the process was repeated with a next cell. The maximum overall injection must not exceed one hour. After this timespan, the PBS was replaced with pre-warmed DMEM and the dish was transferred to the incubator for the next 24 hours.

## Additional Information

**How to cite this article**: Simonis, M. *et al*. Survival rate of eukaryotic cells following electrophoretic nanoinjection. *Sci. Rep.* **7**, 41277; doi: 10.1038/srep41277 (2017).

**Publisher's note:** Springer Nature remains neutral with regard to jurisdictional claims in published maps and institutional affiliations.

## Supplementary Material

Supplementary Information

Supplementary Movie S1
