# Supplementary material for: Survival rate of eukaryotic cells following electrophoretic nanoinjection
Source: Sci Rep. 2017 Jan 25;7:41277. doi: 10.1038/srep41277 (PMC5264641; doi:10.1038/srep41277)
Supplement: Supplementary Information [file srep41277-s1.pdf]

Survival rate of eukaryotic cells following electrophoretic nanoinjection

Matthias Simonis[1], Wolfgang Hübner[1], Alice Wilking[1], Thomas Huser[1,2], and Simon Hennig[1]

[1] Biomolecular Photonics, Department of Physics, University of Bielefeld, Universitätsstr. 25, 33615 Bielefeld, Germany

[2] Department of Internal Medicine and NSF Center for Biophotonics, University of California, Davis, Sacramento, CA 95817, USA

Supplementary Information

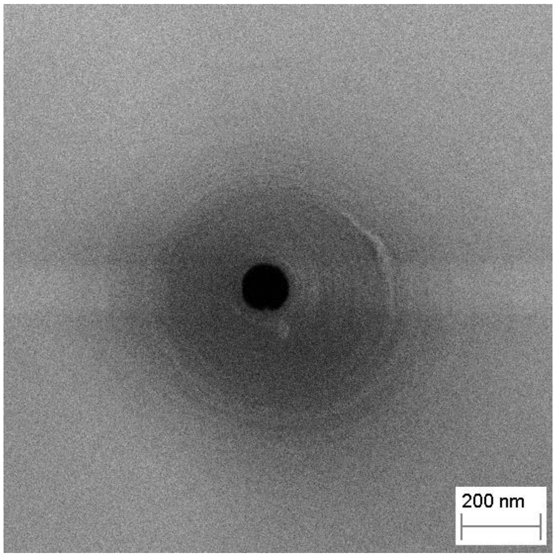

Figure S1: SEM image of a typical nanopipette used for the experiments. The front view into the tip opening (black circle) reveals a tip diameter of 109 nm. Due to the pulling process some variations in the tip diameter of about ±10% are possible. Thus we estimate the average tip diameter of a typical nanopipette to 100 nm.

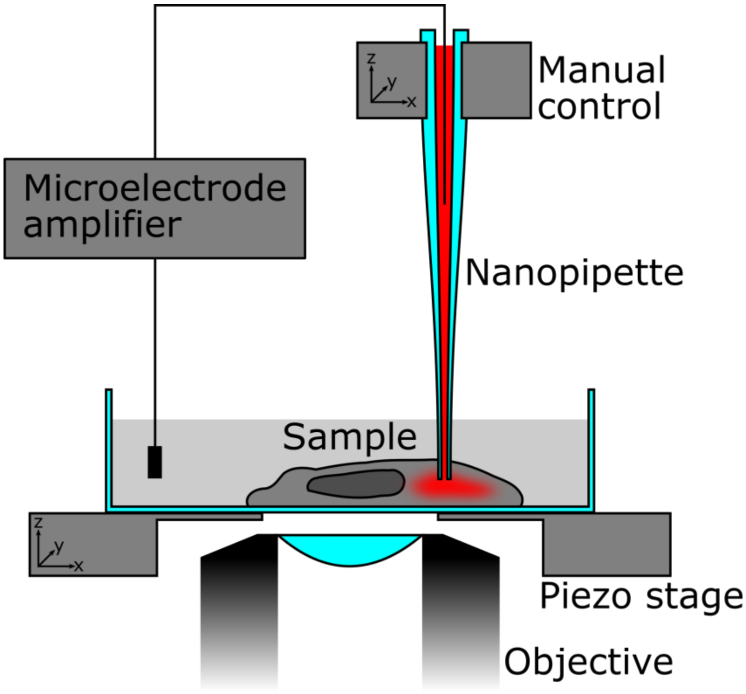

Figure S2: Nanoinjection Scheme. The nanopipette is directly attached to the inverted microscope by a manual 3D control. After manual positioning of the nanopipette the piezostage will move the sample towards the tip of the nanopipette while monitoring the ionic current via the microelectrode amplifier. After penetration, the ejection of molecules begins.

Expermient conditions	Experiment Number	N	dead	alive
100nm, 1 minute, 0.5V, nucleus, no fluorophore	1	18	0	18
	2	14	1	13
100nm, 5 minutes, 0.5V, nucleus, no fluorophore	3	8	1	7
	4	9	2	7
100nm, 1 minute, 1V, nucleus, no fluorophore	5	11	0	11
	6	12	2	10
	7	12	0	12
100nm, 5 minutes, 0.5V, nucleus, no fluorophore	8	5	2	3
	9	9	6	3
	10	6	3	3
	11	6	3	3
100nm, 1 - 10 seconds, 1V, cytoplasm, DAF	12	14	1	13
	13	15	3	12
	14	18	2	16
	15	21	3	18
100nm, 1 - 10 seconds, 1V, nucleus, DAF	16	10	2	8
	17	24	5	19
	18	17	2	15
	19	20	5	15
500nm, 1 - 10 seconds, 1V, cytoplasm, DAF	20	11	7	4
	21	14	7	7
	22	11	9	2
	23	14	8	6
500nm, 1 - 10 seconds, 1V, nucleus, DAF	24	18	11	7
	25	18	14	4
	26	14	8	6

Table S1: Summary of survival experiments. Experiment conditions from left to right: Used pipette tip diameter, duration of injection, applied voltage, target of injection, injected probe. N, number of injected cells. Dead/alive status is checked 24 hours after injection.

Movie S1:

Time-lapse images from Figure 2 of living U2OS cells injected with Dextran - Alexa Fluor 647 (DAF).
